# The Impact of Child Disability on Parental Outcomes: Evidence From Sweden

**DOI:** 10.1002/hec.70017

**Published:** 2025-07-14

**Authors:** Derek Asuman, Ulf‐G. Gerdtham, Ann I. Alriksson‐Schmidt, Martin Nordin, Johan Jarl

**Affiliations:** ^1^ Department of Public Health Aarhus University Aarhus Denmark; ^2^ Department of Economics University of Iceland Reykjavik Iceland; ^3^ Department of Clinical Sciences (Malmo), Health Economics Lund University Lund Sweden; ^4^ Department of Economics Lund University Lund Sweden; ^5^ Centre for Economic Demography Lund University Lund Sweden; ^6^ Department of Clinical Sciences Lund, Orthopaedics Lund University Lund Sweden

**Keywords:** cerebral palsy, children, disability, earnings, employment, event study, labor market, Sweden

## Abstract

Parents of children with disabilities may face higher labor‐market penalties given the extra care and support required. Using Swedish administrative data, we focus on first‐born children with Cerebral Palsy (CP) to estimate impacts on parental labor‐market outcomes. We apply an event study approach to identify effects up to 10 years after the birth of the child. Our results show that employment and earnings of mothers decrease in the short run and increase in the long run whereas for fathers, a marginal decrease is observed in the short run. The effects differ by severity of the disability, with mothers of children with severe impairments driving the increases in the long run, whilst mothers of children with mild impairments appear to experience a penalty. Further, transfers and benefits from the Swedish social insurance system compensate parents for some of the potential costs associated with caring for a child with CP.

## 1 Introduction

A large body of literature has shown that having children worsens parental labor‐market outcomes, especially for mothers (Lundborg et al. 2017; Kleven et al. 2019; Sieppi and Pehkonen 2019; P. Rosenbaum 2021; Markussen and Strøm 2022), although the effect may fade in the long run (Lundborg et al. 2024). These so‐called child penalties account for a large fraction of the gender inequalities in the labor markets in developed countries (Kleven et al. 2019). The causes of these child penalties are still debated. For example, Goldin (2014) and Azmat et al. (2022) argue that the arrival of children pushes parents into jobs characterized by high substitutability and low job uniqueness, typically resulting in lower wages. Andresen and Nix (2022) on the other hand, highlight preference for childcare, gender norms, and discrimination as potential causes of child penalties.

According to the World Health Organization, about 6% of children are born with congenital disabilities each year worldwide.^1^ Children with disabilities require more care, support, and attention than typically developing children. Consequently, parents may stay out of the labor force for a prolonged period or take jobs with greater flexibility to be able to attend to the needs of the child. On the one hand, prolonged labor market disruptions, as well as unpredictable work absence may hurt future employment and earnings prospects of parents of children with disabilities and thus increase the child penalty (Goldin 2014). On the other hand, parents of children with disabilities may increase their labor market activities in order to offset the extra costs of caring for a child with a disability (Becker 1965). As a result, parents of children with disabilities may experience increased stress, physical and mental fatigue, as well as reduced quality of life and life satisfaction (Aktan et al. 2020; Farajzadeh et al. 2020; Fritz and Sewell‐Roberts 2020).

Despite potentially substantial economic effects of parenting a child with disability, there is a paucity of evidence on the parental labor market responses. Most of the available evidence has used cross‐sectional data, focusing on a single point in time. More recently, there has been an increase in the use of longitudinal data to examine the long‐run effects. Further, most studies have focused on mothers, paying little attention to the consequences of having a child with disability on the labor market responses of fathers.

Parental labor market disruptions due to having a child with a disability may negatively affect the circumstances of the child. Households may experience reduced income and financial security, making the family susceptible to financial hardship and negatively affecting the development of the child (Stabile and Allin 2012). It is therefore important to have a family perspective, especially as the impact of having a child with a disability may vary between fathers and mothers, as the latter tend to be the main caregiver (Eriksson 2019).

In this paper, we use rich data from Swedish administrative registers to examine the impact of having a child with Cerebral Palsy (CP), one of the most common lifelong early‐onset musculoskeletal disability, on parental labor‐market outcomes. CP is a neurodevelopmental disability characterized by impairments in posture, muscle tone, and movement, often accompanied by comorbidities and secondary conditions. The motor disorder and associated impairments may interact with social and environmental factors, limiting the child's participation in various life activities.

The focus on CP is interesting from both empirical and policy viewpoints. From an empirical perspective, several challenges exist in isolating the impact of child disability on parental outcomes. First, parents who are more proactive in seeking a diagnosis for their child may differ systematically in their characteristics, potentially biasing estimates. Second, if a disability can be detected in utero, some parents may select to terminate the pregnancy, violating the no‐anticipation assumption. Third, a child's health status at birth is correlated with parental health and socioeconomic status.

CP offers unique clinical and epidemiological advantages for addressing these issues. First, CP is a visible disability, making it unlikely that any child with CP will go undiagnosed. In addition, Sweden's universal healthcare system ensures free access to diagnosis, treatment, medication, and follow‐ups for children, reducing the likelihood of diagnosis bias based on parental socioeconomic status. Secondly, CP can not be detected in utero> and thus we avoid the problem of anticipatory behavior of parents such as pregnancy termination. Thirdly, we show in this paper that parental characteristics in the year before the child's birth are uncorrelated with the likelihood of having a child with CP. These factors collectively allow us to leverage the plausible exogeneity of CP to examine its impact on parental labor market outcomes. For greter precision, we estimate these impacts separately for mothers and fathers, focusing specifically on first births to avoid the potential endogeneity of subsequent births.

From a policy perspective, understanding the labor market consequences of having a child with disability is critical for the design of policies that support parents and children, and to ensure that children with disabilities live in secure and supported environments. Different childhood disabilities manifest in different ways, and thus the consequences on parents will vary accordingly. This paper is distinct from other studies such as Gunnsteinsson and Steingrimsdottir (2019) that focused on a broad definition of childhood disability, thus masking heterogeneity in childhood disability across type, onset, and severity. By focusing specifically on CP, we shed light on the spillover effects of the most common movement disorder among children (Carr and Coghill 2016).

Further, parental responses to permanent disabilities may differ significantly from other health conditions. Öhman et al. (2021) and Adhvaryu et al. (2023) focused on childhood cancers that are remissible in high income countries, and as such the impact on parents may be transitory. Eriksen et al. (2021) on the other hand examined the impact of childhood type‐1 diabetes. CP may be considered a severe condition compared to diabetes, as children with diabetes may rely on insulin to stabilize their conditions. As such, care demands on parents may differ substantially between CP and diabetes, and thus the effect on parental labor‐market outcomes.

Despite similarities in cultural norms, attitudes, and institutional settings across Nordic countries, variations in the organization of social support for persons with disabilities can lead to differences in parental responses to health shocks (Alriksson‐Schmidt et al. 2021). As such, parental response to a child health shock may differ across the countries based on the support available to parents. Children with disabilities and other health conditions may qualify for care benefits, including personal assistance, from the Swedish social insurance system to support activity of daily living in a more independent manner. Access to such personal assistance may ease the care demands on parents and thus affect their labor‐market outcomes. On the other hand, this may offer an opportunity for some parents to become paid personal assistants for their children, especially when the child may require constant care and supervision. Parents who work as personal assistants to their children within the Swedish system are considered regularly employed with contracts and income. However, access to personal assistance is highly correlated with the severity of impairments (von Granitz et al. 2017). There is substantial individual variation in function among persons with CP (P. Rosenbaum et al. 2007), while some children can function independently, others depend entirely on help in basic activities of daily life (Andrén and Grimby 2004). We therefore categorize children with CP by severity of impairment, which enables us to examine differences in parental responses and shed some light on the potential role of the social insurance system on the parental labor‐market consequences of having a child with CP.

Understanding the mechanisms through which having a child with CP may affect parental labor‐market outcomes is crucial. Parents of children with disabilities may shift to flexible jobs in response to having a child with a disability (Eriksen et al. 2021). In the case of Sweden particularly, some parents may choose to be full‐time employed as the paid personal assistance for the child or moonlight as the personal assistant in addition to other jobs. Another factor is the physical and mental burden of caregiving that may lead to fatigue, stress, and burn‐out, increasing the likelihood of sickness absence among parents (Aktan et al. 2020). Parents of children with disabilities face labor‐market disruptions due to long‐run work absence, which may negatively affect their labor‐market outcomes (Markussen 2012). Another channel could be through the effect of having additional children. Müller et al. (2022) reports that parents of children with disabilities are less likely to have additional children. Thus, labor‐market consequences of having a child with a disability may be due to differences in fertility choices between parents.

The objectives of this paper are four‐fold. First, we trace the trajectories of parental labor‐market outcomes in response to having a child with CP. Second, we examine how the Swedish welfare system offsets the impacts of having a child with CP. Third, we explore heterogeneities along severity of impairments and parental characteristics in parental responses to having a child with CP. Finally, we shed light on potential mechanisms through which having a child with CP may affect parental labor outcomes.

Our paper is closely related to recent studies by Michelsen et al. (2015), Gunnsteinsson and Steingrimsdottir (2019), Eriksen et al. (2021) and Adhvaryu et al. (2023) that have examined various childhood health shocks using Danish administrative data, and Breivik and Costa‐Ramón (2024) who used Norwegian and Finnish administrative registers. These studies have reported negative effects of child health shocks on parental labor‐market outcomes, with larger effects for mothers compared to fathers. Using childhood cancers in Sweden, Öhman et al. (2021) finds that the trajectories of parental earnings in response to having a child diagnosed with cancer differ between mothers and fathers. The earnings of fathers decrease after the child is diagnosed with cancer and does not recover to its pre‐diagnosis level within 10 years after diagnosis. However, mothers' earnings decline in the short‐run, and increase in the long‐run compared to mothers of children without cancer.

Our findings reveal that the labor market impact of raising a child with CP also differs between mothers and fathers. While parental employment and earnings decrease in the short run, maternal labor‐market outcomes increase in the long run. We find that the Swedish social insurance system offset some parts of the disability‐related expenses of caring for a child with CP through transfers and benefits to parents. We also observe heterogeneous effects based on the severity of the child's impairments. Mothers of children with severe CP are primarily responsible for the long‐term increase in maternal labor‐market outcomes, while mothers of children with mild CP face a long‐term earnings penalty. These findings highlight the need to pay attention to heterogeneity among persons with disabilities to better understand the differential effects of caregiving on parental labor‐market outcomes.

The remainder of the paper is structured as follows. Section 2 provides a background discussion on the etiology of CP and the setting for the study. Section 3 presents the data and details of variable construction, as well as descriptive statistics. Section 4 details the estimation strategies adopted for this paper. Section 5 presents the main results, heterogeneity analysis by severity of impairments, and potential mechanisms through which having a child with an early‐onset disability may affect parental labor‐market outcomes. We conclude the paper with a discussion of the results and how the findings can be understood in Section 6.

## 2 Background

### 2.1 Etiology of CP

CP is the most prevalent early‐onset permanent motor disability and affects 2–3 per 1000 live births in OECD countries (Kirby et al. 2011; Smithers‐Sheedy et al. 2014). Recent estimates, however, report decreasing prevalence across Europe (Sellier et al. 2016; Hollung et al. 2018; Larsen et al. 2021; McIntyre et al. 2022), Canada (Robertson et al. 2017), Japan (Touyama et al. 2016), Australia (Galea et al. 2019), and China (He et al. 2017). In Sweden, Himmelmann and Uvebrant (2018) estimates the prevalence of CP to be 2 per 1000 live births between 2007 and 2010. CP is caused by a non‐progressive brain damage that occurs in the developing fetal or infant brain (P. Rosenbaum et al. 2007; Kirby et al. 2011). CP is often associated with comorbidities and secondary conditions such as epilepsy and musculoskeletal problems that generally worsen over time. Symptoms of CP may appear in the first few months after birth, however, diagnosis may occur later. In Sweden, the diagnosis of CP typically occurs at 4 years of age.

The etiology of CP is complex and oftentimes uncertain, and the underlying causes are poorly understood (McIntyre et al. 2013; Korzeniewski et al. 2018). CP is often considered to result from several predisposing factors (Himmelmann and Uvebrant 2018; Korzeniewski et al. 2018). Previous researchers have identified a number of potential antenatal, neonatal, and postnatal risk factors of CP. The most prominent include congenital brain malformation, complications and infections during pregnancy and delivery, pre‐term, low birth weight, low gestational age at delivery, and multiple gestation. Some studies have identified socioeconomic characteristics such as low maternal age at birth, educational attainment, multiple pregnancies, and low socioeconomic status to be correlated with an increased risk of CP (Korzeniewski et al. 2018; Michael‐Asalu et al. 2019).

### 2.2 Study Setting

Sweden, like other Scandinavian countries, is well known for egalitarian gender attitudes and a comprehensive welfare system. Family‐friendly policies, such as subsidized day‐care and paid job‐protected parental leave, offer opportunities for family and work balance. In addition, health insurance coverage is tax‐funded and universal with an emphasis on equitable access to services and the availability of greatly subsidized medications, treatments, and assistive devices. Thus, a child's health insurance is not tied to parental employment status, implying the absence of a health insurance motive in parental labor market responses.

Parental leave has been available to both parents in Sweden since 1974, when parents were eligible for 6 months of paid parental leave. While mothers historically take most of the leave days, the share taken by fathers has steadily increased over time (Rosenqvist 2024). Currently, parents are eligible for 480 days of parental leave, to be used before the child turns 12 years of age. Of these, 390 days are salary‐based with a replacement rate of up to 80% (capped at SEK 37,500SEK (EUR 3750) per month). The remaining 90 days are paid at a low flat rate. For multiple birth, parents receive additional 180 days of leave for each additional child. A mother is entitled to parental leave from 60 days before the expected birth. Both parents are also entitled to parental leave to participate in parental training when expecting a child. From the time the child is born, parental leave is paid to the parent who is at home with the child. In addition to parental leave, parents of children older than 8 months and younger 12 years can receive a up to 120 days of temporary parental leave for child care if the parent has to stay home from work or misses unemployment benefits to care for a child due to illness.

Children with disabilities, as well as their parents and legal guardians, may also receive additional benefits. These benefits are covered under the Act Concerning Support and Service for Persons with Certain Functional Impairments (LSS) and apply to individuals with considerable or permanent functional impairments to ensure the rights to basic measures (Alriksson‐Schmidt et al. 2021). Benefits are not dependent on the financial means of the family (or caregivers), but on the needs of the child. A child covered by LSS can receive personal assistance with the initial 20 h of assistance per week paid for by the municipality, and if additional assistance is required, this is covered by the Swedish Social Insurance Agency (SSIA).

Parents can also apply for childcare allowance for care and supervision from SSIA if a child under 19 years of age with disability needs long‐term care or supervision. This is a benefit separate from personal assistance but can only be granted for care not covered by personal assistance. It is based on the need for care and supervision of the child and is unconditioned on parental labor market status. Further, parents can apply for an allowance for extra cost to offset major disability‐specific expenses such as assistive devices and modification of residence, and in the event of severe mobility difficulties, parents may be granted a car allowance to purchase or adapt a car or other vehicle suitable to accommodate the person with a disability. Parents can also receive 10 contact days per year per child with disability to learn how to support the child, as well as respite care and relay services where a temporary caretaker cares for the child.

## 3 Data, Variables and Descriptive Statistics

### 3.1 Data and Sample Construction

The data for this study are constructed from several Swedish administrative registers. The registers are linked using unique person identification numbers. We identified all individuals with CP and living in Sweden between 1990 and 2015 using the diagnostic ICD 10 code G80 from the National Quality Register and Follow‐up Program for Individuals with Cerebral Palsy (CPUP), the National Patient Register, and the Medical Birth Register. We exclude individuals who do not have a diagnosis of CP after the age of 4 years. Individuals with acquired brain damage^2^ after the age of two, but no CP diagnosis before the brain damage are also excluded. Further, individuals are excluded if they have other diagnoses^3^ that are considered incompatible with CP. Finally, individuals who have been written off in the CPUP register are excluded from the sample.

We generate a comparison group of individuals without CP based on sex, year of birth and municipality of residence using the Register of the Total Population. Siblings of individuals with CP are excluded from the comparison group to avoid intra‐family spill‐over effects. We then match children to their parents in both groups using the Multi‐generation Register, which contains information on familial relationships.

We extract information on labor market participation from the Longitudinal Integrated Database for Health Insurance and Labor Market Studies (LISA) database. LISA is a longitudinal database comprising detailed data on the social insurance at the individual level, including parental‐ and unemployment insurances. We observe labor market status, earnings, and other incomes, including income from social insurance separate for each type of benefit. Demographic information is drawn from the Register of the Total Population and information on inpatient care use is obtained from the Patient Register.

We construct a balanced panel of parents who are observed 5 years before the event of childbirth to 10 years after. For this study, we limit the sample to parents of children born between 1995 and 2010 in Sweden who survived at least 10 years after birth. To minimize endogeneity related to future births, we restrict the analysis to mothers' firstborn children. We exclude parents with non‐singleton births from the sample. For fathers, we are unable to identify whether the child with CP is the first birth. The treatment group in this study consists of parents of children with CP whilst the control group consists of parents of children without CP or spina bifida. We estimate the effects separately for mothers and fathers. Our final sample includes 6778 matched mother‐child pairs, of which 1014 (15%) are mothers of children with CP, and 6778 father‐child paired matches of which 1012 (15%) are fathers of children with CP.

In this paper, we define employment as a binary outcome, constructing an indicator equal to one if an individual was employed at any time during the year and zero otherwise. Earnings is defined as all earnings from employment and self‐employment that are reported to the Swedish Tax Agency, conditioned on employment. Previous studies have shown that earnings conditioned on being employed with an annual gross earnings exceeding SEK 100,000^4^ (EUR 10,000) produces results that are similar to hourly wages when using Swedish administrative data (Dackehag et al. 2015; Lundborg et al. 2015; Lovén et al. 2017). This threshold is equivalent to about 3 months of full‐time employment at the average salary in 2015 and ensures that we consider only individuals with strong attachment to the labor market. We apply this threshold to test the robustness of our results and follow Kleven et al. (2019) and P. Rosenbaum (2021) and use the levels instead of the logarithm in the estimations.

In addition to employment and earnings, we examine the impact of having a child with CP on parental disposable income. We do not have information on parental consumption expenditure or asset accumulation that would enable us to examine the impact on parental welfare, financial status, or disability‐specific expenditures. However, parental disposable income gives us an indication of the financial resources available to parents. Disposable income is defined as total income received (including allowances and benefits) minus taxes paid and is measured at the individual level.

### 3.2 Descriptive Statistics

Table 1 compares the characteristics of parents of children with CP with the comparison group of parents of children without CP. Mothers appear to be statistically comparable in many characteristics ‐ background, education, married or cohabiting ‐ 1 year before the birth of the child. The exception is age where mothers of children with CP are 0.6 years older, and employment where they are about 1.5% points less likely to be employed. Fathers of children with CP are 0.5 years older, 5% points more likely to have completed secondary education, and 3% points less likely to have higher education compared to the fathers in the comparison group. Whilst 56% of mothers are employed in the private sector, 86% of fathers were employed in the private sector the year before the birth of the child.

**TABLE 1:** Parental characteristics 1‐year before birth.

	Mothers				Fathers			
Variables	All	With CP	Without CP	Diff.	All	With CP	Without CP	Diff.
Age	27.72	28.24	27.62	0.61***	30.41	30.85	30.33	0.51***
Married/cohabiting (%)	28.40	27.43	28.58	−1.15	29.53	27.58	29.87	−2.29
Background (%)								
Swedish	80.43	80.20	80.47	−0.27	77.94	78.37	77.87	0.50
Foreign born	7.76	8.61	7.61	−1.00	10.32	9.42	10.48	−1.05
Domestic born, foreign	11.81	11.19	11.92	−0.73	11.74	12.20	11.66	0.54
Education (%)								
Mandatory	9.12	8.91	9.15	−0.24	12.86	11.61	13.08	−1.47
Secondary	51.79	53.76	51.44	−2.32	55.28	59.33	54.59	4.74***
Higher	39.09	37.33	39.40	2.07	31.85	29.07	32.33	−3.27**
Employment (%)								
During the year	94.45	93.07	94.69	−1.62**	94.19	94.25	94.18	0.06
Earn ≥ SEK 100,000	75.28	72.77	75.72	−1.47**	78.61	79.07	78.53	0.05
Private sector	56.59	54.79	56.90	−2.11	85.14	85.89	85.01	0.89
Earnings and income (1000 SEK)								
Earnings	188.53	184.08	189.31	−5.23	246.45	247.68	246.24	1.43
Disposable income	154.60	152.89	154.90	−2.01	191.10	192.86	190.79	2.06
Sickness absence (days)	10.14	10.6	11.09	0.49	4.98	4.56	5.05	0.49

*Note:* All characteristics are measured 1 year prior to birth of the child to avoid the characteristics being impacted by childbirth and CP diagnosis. All monetary values are reported in 1000 SEK adjusted to 2015 level using the CPI. 10 SEK ≈ EUR 1.

*p < 0.1.

**p < 0.05.

***p < 0.01.

Testing multiple outcomes as shown in Table 1, increases the risk of type‐1 errors. To address this challenge, we perform a multiple hypothesis correction based on the approach proposed by Romano and Wolf (2016) to the difference test. Our results do not show systematic differences between parents prior to the birth of a child with CP (see Table 2). This further supports the assumption that having a child with CP is exogenous to parental characteristics in Sweden.

**TABLE 2:** Adjusted *p‐values* for multiple hypothesis testing.

Outcome	Mothers		Fathers	
	Model *p*‐value	RW *p*‐value	Model *p*‐value	RW *p*‐value
Age	0.0001	0.002	0.009	0.454
Married/cohabiting	0.455	0.953	0.141	0.784
Swedish background	0.839	0.975	0.721	0.997
Foreign born	0.269	0.864	0.311	0.926
Domestic born, foreign	0.506	0.953	0.619	0.997
Mandatory education	0.804	0.975	0.198	0.827
Secondary education	0.174	0.830	0.005	0.454
Higher education	0.213	0.864	0.040	0.454
Employed during the year	0.038	0.363	0.934	0.997
Earn ≥ SEK 100,000	0.045	0.391	0.703	0.997
Private sector	0.227	0.864	0.477	0.992
Earnings	0.190	0.844	0.812	0.997
Disposable income	0.389	0.864	0.573	0.986
Sickness absence	0.696	0.975	0.644	0.997

*Note:* This table shows the *p*‐values associated with the estimated differences between parents of children with CP and the comparison 1 year before the birth of the child. The RW *p*‐value adjusts for multiple hypothesis testing using the Romano and Wolf (2016) correction with 1000 replications.

**FIGURE 1:**
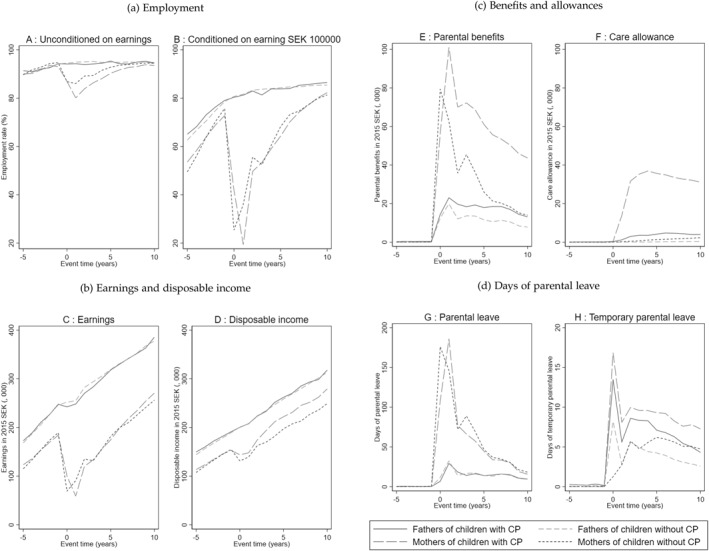
Labor market trajectories of parents. The figure shows the trajectories of parental outcomes 5 years prior to and 10 years after the birth of the child. All monetary values are reported in 1000 SEK adjusted to 2015 level using the CPI. The trajectories are crude means for the group of parents. Event time = 0 is the year of birth of the child.

We also plot the crude average outcomes from 5 years prior to the birth of the child to 10 years after the birth in Figure 1. The figure shows a level difference between fathers and mothers, except for employment at the extensive margin. The figure also shows that before the birth of the child, the trajectories of the outcomes were parallel. We find large differences between fathers and mothers immediately after the birth of the child. Whereas fathers' labor‐market outcomes seem to remain unchanged or change marginally, mothers, on the other hand, experience large declines in labor‐market outcomes. While mothers of typically developing children experience the largest declines in outcome in the year of birth of the child, mothers of children with CP experience the largest decline 1 year after the birth of the child, when mothers of typically developing children are likely to have started to return to the labor market. Further, mothers take significantly more parental leave days than fathers as shown in Panel G of Figure 1d.

## 4 Estimation Strategy

We empirically examine the effect of having a child with CP on parental labor‐market outcomes using an event study framework. This approach has been previously used by Gunnsteinsson and Steingrimsdottir (2019), Eriksen et al. (2021) and Adhvaryu et al. (2023) to study the impact of the various childhood health shocks on parental outcomes. The event study framework enables us to capture the full dynamic effects of the event over time. Specifically, we estimate the following regression:

(1) $$Y_{\mathrm{irst}}=\sum_{\begin{array}{c} j=-5\\ j\neq -1 \end{array}}^{+10}\alpha_{j}1\left(j=t\right)D_{i}+\sum_{\begin{array}{c} j=-5\\ j\neq -1 \end{array}}^{+10}\beta_{j}1\left(j=t\right)+\sum_{i}\gamma_{i}X_{\mathrm{irst}}+\sum_{k}\delta_{k}1\left(k=age_{is}\right)+\sum_{y}\phi_{y}1\left(y=s\right)+\sigma_{r}+\epsilon_{\mathrm{irst}}$$

where $Y_{\mathrm{ist}}$ is the labor market outcome of interest of parent i in region r at calendar year s and event time t. The first term of the equation is a set of event time dummies. Event time is defined as the years relative to the birth of a child and denote the year of birth of the child by t=0. We omit event time t=−1, so that the coefficients for the other time dummies measure the effect relative to 1 year before the birth. $D_{i}$ is an indicator variable which equals to 1 if the parent has a child with CP. The $\alpha_{j}$ coefficients are estimates of the effect of having a child with disability, that is, the difference between parents in the treated and control groups, the average treatment effect on the treated (ATT). The event of having a child with CP mostly coincides with the timing of birth. Parental labor market responses to having a child with CP may be conflated by the effects of childbirth, which is associated with substantial labor market penalties especially for mothers (Kleven et al. 2019; P. Rosenbaum 2021). $\beta_{j}$ estimates the coefficients of having a child on labor‐market outcomes (child penalties). $\alpha_{j}+\beta_{j}$ represents the coefficients of the treatment group. In our results, we plot $\alpha_{j}$ for selected labor‐market outcomes.

$X_{i}$ is a set of background characteristics included as controls to account for potential cofounding factors. It also includes parental labor‐market outcome in the reference year to account potential differences in labor‐market trajectories. We include a full set of age dummies (δ) to control for underlying non‐parametric trends in parents labor‐market outcomes, and calendar year dummies (ϕ) to take into account year‐specific fluctuations. We also include region of residence dummies (σ) to capture regional differences in labor market conditions. The error term $\left(\epsilon_{\mathrm{irst}}\right)$ captures any unexplained variations. We estimate these models by OLS and cluster standard errors at individual level to adjust for correlations across years.

The interpretation of our results as the casual impact of having a child with CP on parental labor‐market outcomes is conditioned on the assumption that unobservable factors that cause CP are uncorrelated with parental labor‐market outcomes. For the identification assumption to hold, having a child with CP should be random. The assumption of strict exogeneity implies that parents in the treated and control groups must be comparable before the birth of the child, and therefore should be equivalent in potential later life outcomes. Finally, there should be no anticipatory behavior among parents with respect to having a child with CP.

Although a small fraction of CP is caused by postnatal events, Korzeniewski et al. (2018) argue that most of the factors predisposing children to CP are no longer prevalent in high income countries. In a recent study, Müller et al. (2022) show that the predictors of CP in Sweden are largely unknown based on data extracted from administrative registers. We test whether parental characteristics in the year before the child's birth predict the likelihood of CP. Specifically, we control for educational attainment, disposable income, immigrant status, married/cohabiting status, age, and employment status.

The results are shown in Table 3. In Column (1), the sample includes all mothers whilst Column (2) shows the results for fathers. In Column (3), we replace maternal education and disposable income with the highest parental education and total disposable income of both parents. Our results show that mothers of children with CP are comparable to mothers of children without CP in our sample whilst fathers differ only in secondary education. These results, together with the pattern Figure 1, suggest that having a child with CP can be treated as an exogenous event in our sample.

**TABLE 3:** Parental characteristics and likelihood of CP.

	1	2	3
	Mothers	Fathers	Parents
Secondary education	0.011	0.033**	0.012
	(0.016)	(0.014)	(0.026)
Higher education	−0.008	0.008	−0.011
	(0.018)	(0.015)	(0.028)
Log. disposable income	−0.002	−0.007	−0.002
	(0.006)	(0.006)	(0.010)
Foreign born	0.011	−0.007	0.009
	(0.017)	(0.016)	(0.017)
Domestic born, foreign bkgd.	−0.007	0.011	−0.007
	(0.013)	(0.014)	(0.013)
Married/cohabiting	−0.010	−0.010	−0.009
	(0.010)	(0.010)	(0.010)
Age	−0.008	0.001	−0.005
	(0.011)	(0.006)	(0.011)
Age squared/100	0.022	0.002	0.019
	(0.019)	(0.009)	(0.019)
Employed	−0.022*	0.009	−0.023*
	(0.012)	(0.013)	(0.012)
Constant	0.244	0.182	0.213
	(0.160)	(0.114)	(0.183)
Observations	6747	6417	6764
*R*‐squared	0.006	0.004	0.006
Year fixed effects	YES	YES	YES

*Note:* For a balance test, we use an (0/1) outcome variable of whether the child is diagnosed with CP and test whether observable characteristics predict the diagnosis separately for mothers and fathers. Employed refers to employment at the intensive margin. In Column (3) we replace maternal education and disposable income with the highest parental education and total parental disposable income. Robust standard errors in parentheses.

*p <0.1.

**p <0.05.

***p <0.01.

## 5 Results

### 5.1 Main Results

#### 5.1.1 Employment and Earnings

**FIGURE 2:**
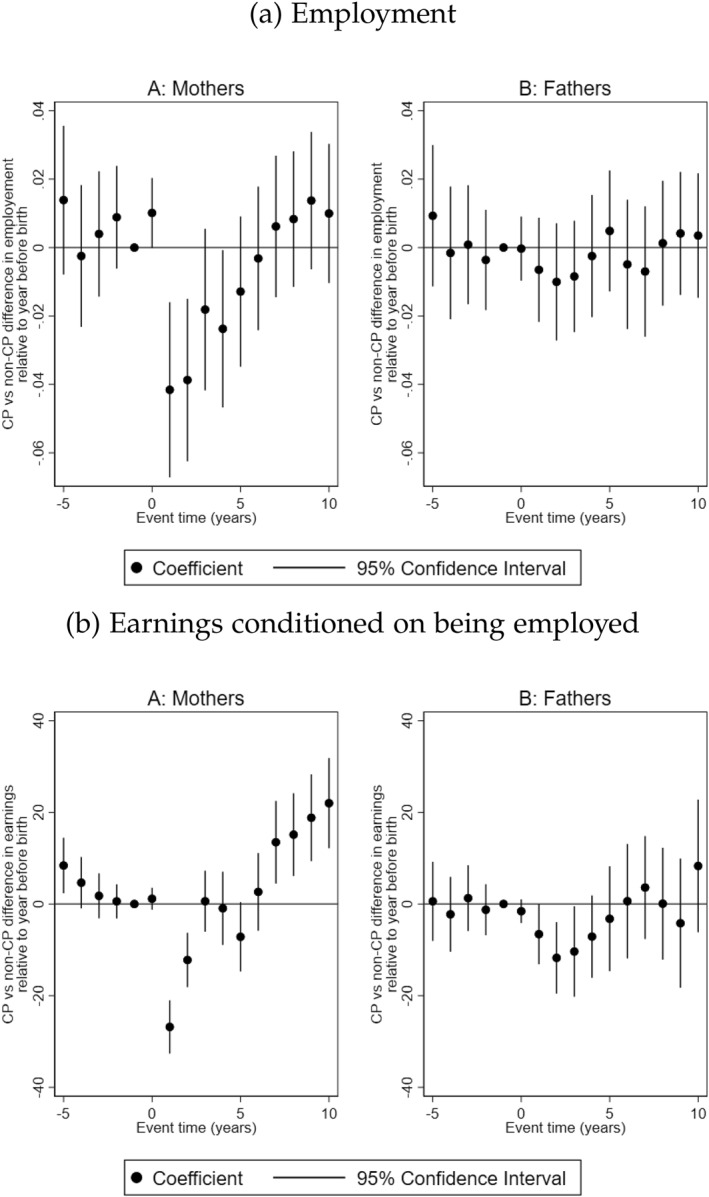
Labor‐market response to having a child with CP. The figure shows the trajectories of the employment and annual earnings conditioned on being employed during the year for mothers and fathers, and 95% confidence intervals. Event time = 0 is the year of birth of the child.

We begin by examining the employment response of parents after having a child with CP. Figure 2a shows that the employment of treated mothers unconditioned on earnings is reduced by about 2%–4% points between the first and fourth years after childbirth. This is equivalent to 2.1%–4.2% drop in employment of treated mothers relative to the employment rate of mothers in the control group in the reference year (1 year before the birth of the child). From the fifth year onward, no statistical differences are observed between the employment rates of treated and comparison mothers. For fathers, there are no statistically significant differences in employment at any point.

Next, we examine the impact on earnings conditioned on being employed. Figure 2b shows that the earnings of parents do not differ prior to the birth of the child. In the first year after the birth of the child, treated mothers earn approximately SEK 27,000 (EUR 2700) less than mothers in the control group, reducing to SEK 12,000 (EUR 1200) less in the second year. In relative terms, this is equivalent to 14% and 6% decrease in the earnings of treated mothers compared to the average earnings of mothers in the control group^5^ in the reference year. From the seventh year after birth, we observe that the difference in earnings turns in favor of treated mothers, with an earning premium of SEK 13,000 (EUR 1300) to SEK 22,000 (EUR 2200). The increase ranges between 6.5% and 11% of the average earnings of mothers in the control group in the reference year. We find that treated fathers earn between SEK 6600 (EUR 660) and SEK 11,800 (EUR 1180) less in the first 3 years after the birth of the child with CP. This is about 2.5%–4.5% of the earnings of fathers in the control group in the reference year.^6^

The impact of having a child with CP on parental labor‐market outcomes, particularly for mothers, is expected to become evident when parents in the comparison group return to the labor market. Although, CP should formally be diagnosed at 4 years of age in Sweden, its symptoms typically manifest earlier. As such, children with CP may require more care compared to children without CP from the first year of life. This increased caregiving burden may lead to that mothers, in particular, delay their return to the labor market following the birth of a child with CP.

**FIGURE 3:**
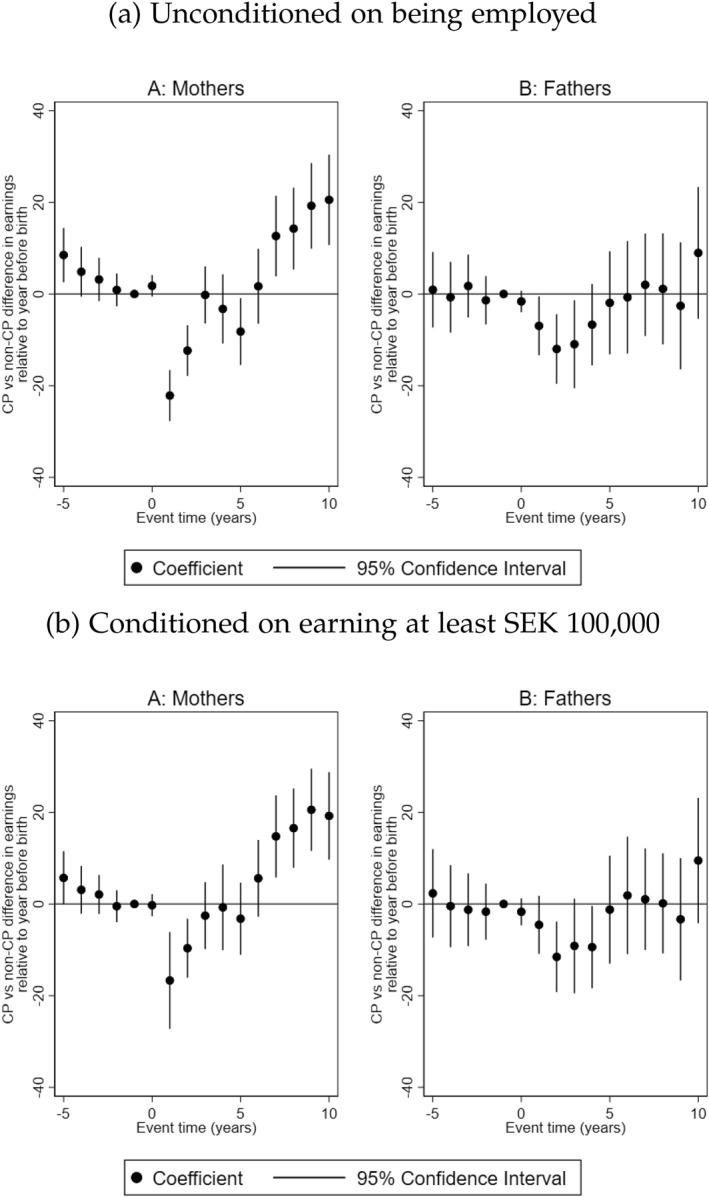
Parental earnings response to having a child with CP. The figure shows the trajectories of earnings for mothers and fathers unconditioned on employment and conditioned on having annual income from employment and self‐employment of at least SEK 100,000, and 95% confidence intervals. All monetary values are reported in 1000 SEK. Event time = 0 is the year of birth of the child.

Our estimates of the impact of having a child with CP on parental earnings is conditioned on being employed at any point in time during the year. This approach may capture only the effects for those employed instead of the entire labor earnings effects (P. Rosenbaum 2021). We therefore test the sensitivity of our results in two ways. First, we estimate earnings unconditioned on being employed during the year. Our results are presented as Figure 3a. We observe similar trends of parental earnings response to having a child with CP as before. Mothers of children with CP earn SEK 22,000 (EUR 2200) and SEK 12,000 (EUR 1200) less in the first 2 years after the birth of the child, and from the seventh year and onward, they earn between SEK 12,600 (EUR 1260) and SEK 20,600 (EUR 2060) more than mothers in the comparison group. This is equivalent to a 12% and 6% decline in the first 2 years and between 7% and 11% increase in earnings from the seventh year after the birth of the child compared to control mothers^7^ in the reference year. Fathers on the other hand observe a drop in earnings in the short‐run after the birth of the child with CP, earning between SEK 7000 (EUR 700) and SEK 12,000 (EUR 1200) less than fathers in the comparison group in the first years after the birth of the child with CP. This is equivalent to a 2%–4% decline in earnings compared to fathers in the control group^8^ in the reference year. Subsequently, the earnings penalty fades for fathers.

Second, we condition earnings on having a strong attachment to the labor market by using a threshold of earning at least SEK 100,000 annually. We observe that mothers of children with CP earn SEK 16,700 (EUR 1670) and SEK 9700 (EUR 970) less in the first and second years after the birth of the child with CP. In the first 2 years, treated mothers face a earnings penalty equivalent to 7% and 4% of the earnings of mothers in the control group^9^ in the reference year. From the seventh year however, the penalty turns in favor of mothers of children with CP, as they earn between SEK 14,700 (EUR 1470) and SEK 20,500 (EUR 2050) more than mothers in the comparison group (see Figure 3b). This is about 6%–9% increase in earnings relative to the earnings of mothers in the control group^9^ in the reference year. For fathers, we observe a decline in earnings of SEK 11,500 (EUR 1150) in the second year after the birth of the child with CP, after which there is no significant difference between fathers. In relative terms, the earnings of treated fathers declined by 4% in the second year compared to the earnings of fathers in the control group in the reference year.^10^

#### 5.1.2 Disposable Income

**FIGURE 4:**
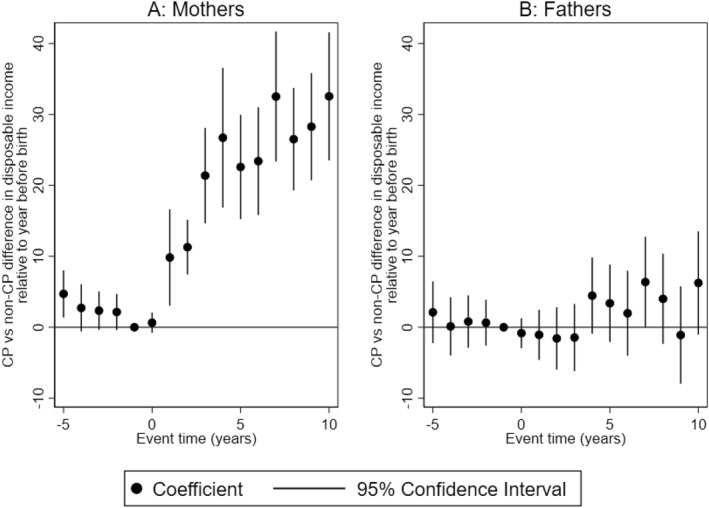
Parental disposable income response to having a child with CP. The figure shows the trajectories of disposable income for mothers and fathers, and 95% confidence intervals. All monetary values are reported in 1000 SEK. Event time = 0 is the year of birth of the child.

In Figure 4, we present parental disposable income response to having a child with CP. We find statistically significant and economically meaningful impacts on mothers. From the second year after birth, treated mothers report higher disposable income compared to mothers in the control group. The difference increases over time, from SEK 10,000 to SEK 32,500 (EUR 1000 to EUR 3250) in the 10th year after the birth of the child. This is equivalent to 6%–21% increase relative to the average disposable income of mothers of children without CP 1‐year before the birth of the child. There is no effect on the disposable income of fathers.

#### 5.1.3 Parental Benefits From Social Insurance

Parents receive a number of benefits from the social insurance system to support childcare needs (see Section 2.2 for a brief overview of the benefits available to parents). We analyze the trajectories of parental benefits from the social insurance system following the birth of the child with CP. First, we assess total income prompted by parental benefits (which is the sum of parental leave income, temporary parental allowance for childcare and care allowance).^11^ We then proceed to examine care allowance specifically, as it is targeted at children with functional impairments.

**FIGURE 5:**
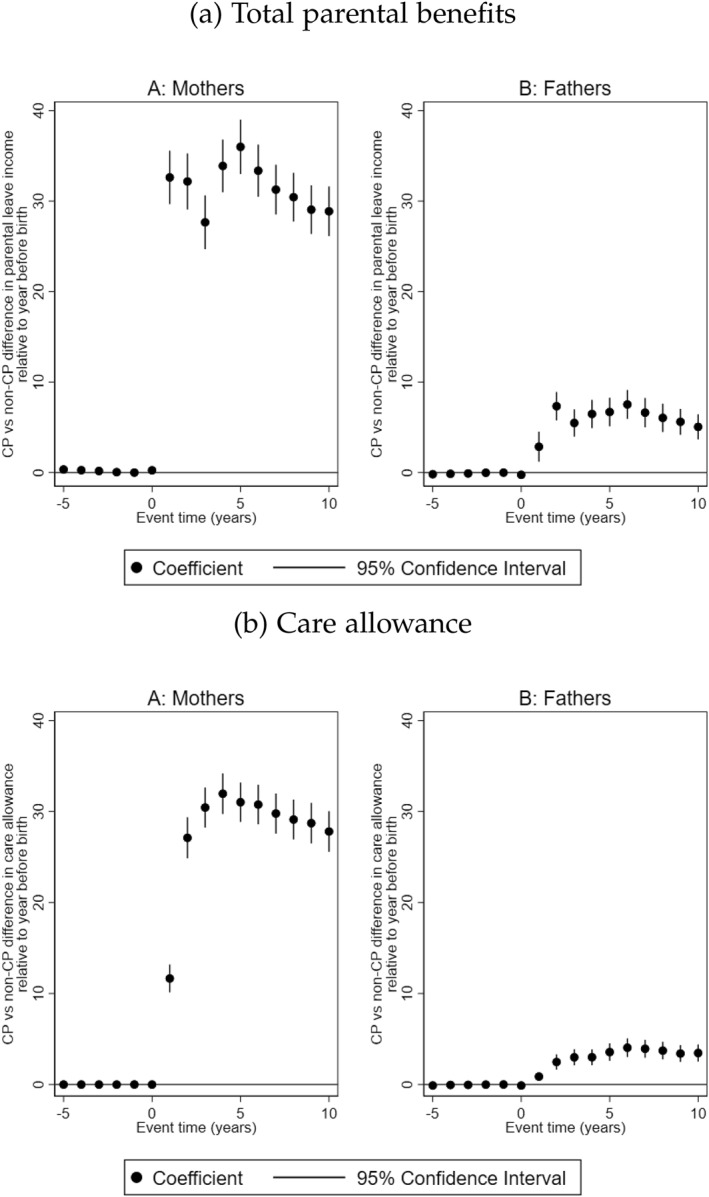
Parental allowance in response to having a child with CP. The figure shows the trajectories of benefits and transfers from the social insurance system to mothers and fathers, and 95% confidence intervals. All monetary values are reported in 1000 SEK. Event time = 0 is the year of birth of the child.

As expected, we do not find differences in parental benefits between parents prior to the birth of the child. In the years after birth, treated mothers received on average SEK 31,500 (EUR 3150) per year more in income prompted by parental benefits than mothers in the control group, whilst treated fathers receive an average parental benefits income of SEK 6000 (EUR 600) per year more than fathers in the control group (Figure 5a). In Figure 5b, we show that treated mothers receive about SEK 11,600 (≈ EUR 1160) in care allowance in the first year after the birth of the child. From the second to tenth year, treated mothers received approximately SEK 30,000 (≈ EUR 3000) per year in care allowance. Treated fathers on the other hand received about SEK 3400 (≈ EUR 340) in care allowances over the same period.

**FIGURE 6:**
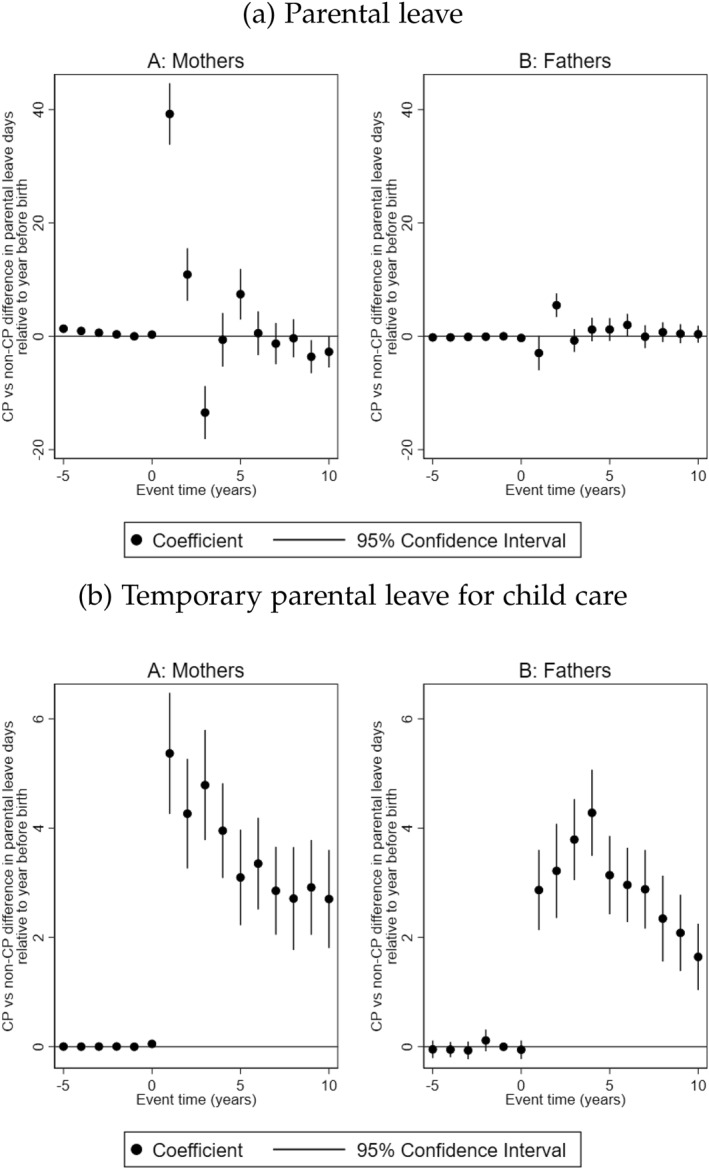
Days of parental leave in response to having a child with CP. The figure shows the trajectories of number of parental leave days for mothers and fathers, and 95% confidence intervals. Event time = 0 is the year of birth of the child.

Income prompted by parental leave is a function of the compensation rate and the number of days taken, as such, differences in parental leave income may reflect differences in the compensation rate between parents. We therefore examine the number of days of leave taken by parents following the birth of a child with CP. We begin by examining the number of days of parental leave (see Figure 6a). We find that treated mothers take 40 and 11 days more parental leave in first and second years after the birth of the child with CP. In the third and fourth years, treated mothers take 13 days less and 7 days more parental leave compared to mothers in the comparison group. For fathers, we find that treated fathers take 5 days more parental leave in the first year after the birth of the child compared to their counterparts in the comparison group. It should be noted that parents, irrespective of the disability status of the child, are entitled 490 days of parental leave that can be used until the child is 12 years old. As such, parents in the comparison group may have saved days of parental leave to be used after the follow‐up period. Parental leave days are to be shared between parents although previous studies have shown that fathers take fewer days of parental leave (Rosenqvist 2024), which is consistent with our findings.

Once parents return to work after childbirth, they are eligible for temporary parental leave for a sick child for up to 60 days per year. We examine the differences in the number of temporary parental leave days taken by parents of children with CP compared to those in the comparison groups (see Figure 6b). Mothers of children with CP take 3–5 days more in temporary parental leave compared to mothers in the comparison group, while fathers of children with CP take 2–4 days more relative to their counterparts in the comparison group. These findings suggest that the observed differences in disposable incomes (Figure 4) between parents of children with and without CP are primarily driven by the care allowance received by parents of children with CP.

### 5.2 Heterogeneity Analysis

#### 5.2.1 Severity of Impairments

The results may mask some heterogeneity along the severity of impairments as responses might differ between parents of children with mild and severe impairments. We therefore repeat our analysis separately for sub‐samples of parents defined by the severity of impairments based on the Gross Motor Function Classification System (GMFCS), a five‐level system that classifies gross motor function based on self‐initiated movement abilities (P. L. Rosenbaum et al. 2008; Palisano et al. 2008). Higher GMFCS levels correspond to greater gross motor functional impairment (Andersen et al. 2008). GMFCS has been shown to be correlated to other classification systems of severity of CP such as the Manual Ability Classification System (MACS) and the Communication Function Classification System (CFCS) (Compagnone et al. 2014).

GMFCS levels I to III correspond to self‐initiated movement with/without the support of a hand‐held device whilst levels IV and V correspond to severe motor impairments that prevent independent functioning and we define the former as mild and the latter as severe CP. Kirby et al. (2011) and Christensen et al. (2014) estimate that about 40% of children with CP cannot walk independently. In cases where GMFCS levels are missing, we define severity based on CP subtype. Jonsson et al. (2019) show that CP subtype is correlated with gross motor function, although the correlation is not clear for all subtypes. In our sample, 642 (63.3%) children with CP are classified as mild and 319 (31.5%) as severe. The remaining 53 (5.2%) cannot be defined by severity with available information. We include only the matched comparisons of each of the severity groups in the stratified analysis.

**FIGURE A1:**
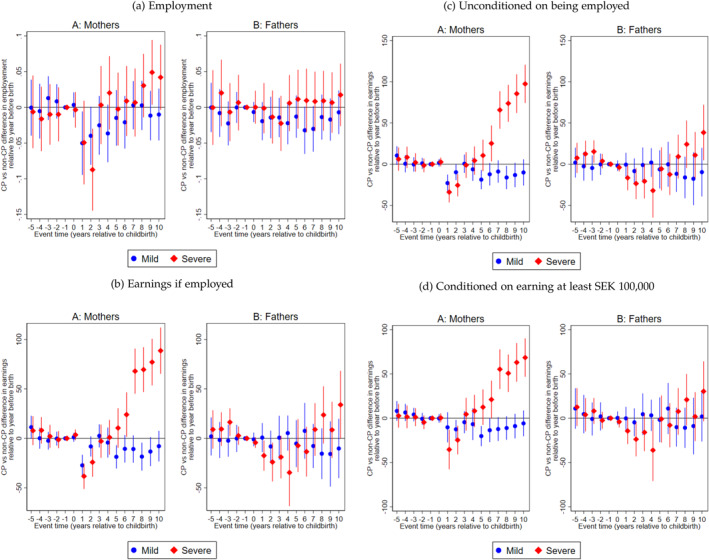
Parental labor‐market response to having a child with CP. The figure shows the trajectories of the employment and annual earnings conditioned on being employed during the year for mothers and fathers, and 95% confidence intervals. Event time = 0 is the year of birth of the child.

The results are available in Appendix A and indicate heterogeneity in parental labor market responses across severity of impairments. In the short‐run, mothers of children with mild and severe impairments both experience a decline in employment and earnings (Figure A1) compared to the control group, though the effects are greater for mothers of children with severe impairments. In the long‐run however, the observed increase in employment and earnings reported in Section 5.1.1 is primarily driven by mothers of children with severe impairments, whilst mothers of children with mild impairments face a earnings penalty. Further, fathers of children with severe impairments show increases in employment and earnings whilst fathers of children with mild impairments remain unaffected.

**FIGURE A2:**
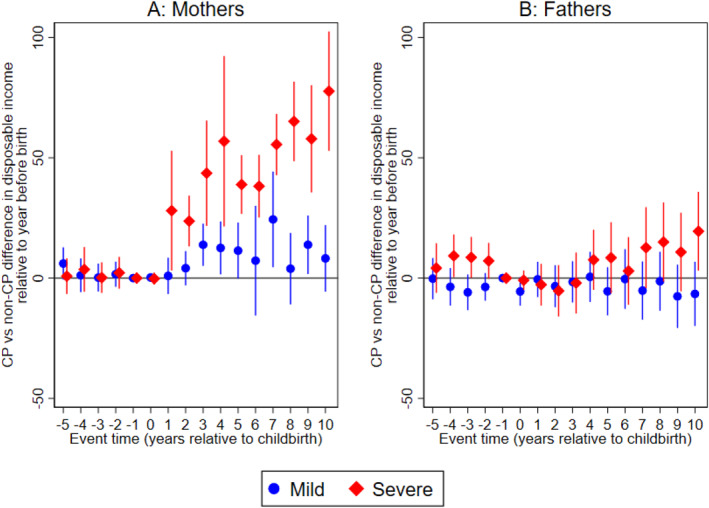
Parental disposable income response to having a child with CP. The figure shows the trajectories of disposable income mothers and fathers, and 95% confidence intervals. All monetary values are reported in 1000 SEK. Event time = 0 is the year of birth of the child.

**FIGURE A3:**
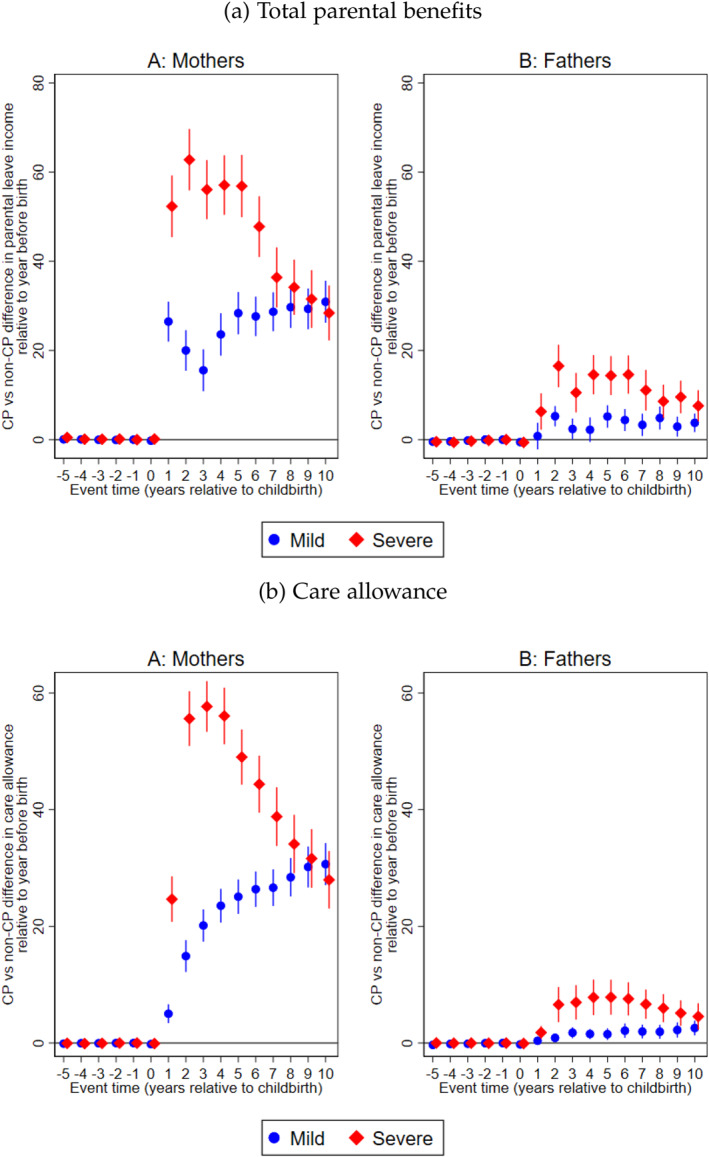
Parental benefits response to having a child with CP. The figure shows the trajectories of parental leave income and care allowance for mothers and fathers, and 95% confidence intervals. All monetary values are reported in 1000 SEK. Event time = 0 is the year of birth of the child.

**FIGURE A4:**
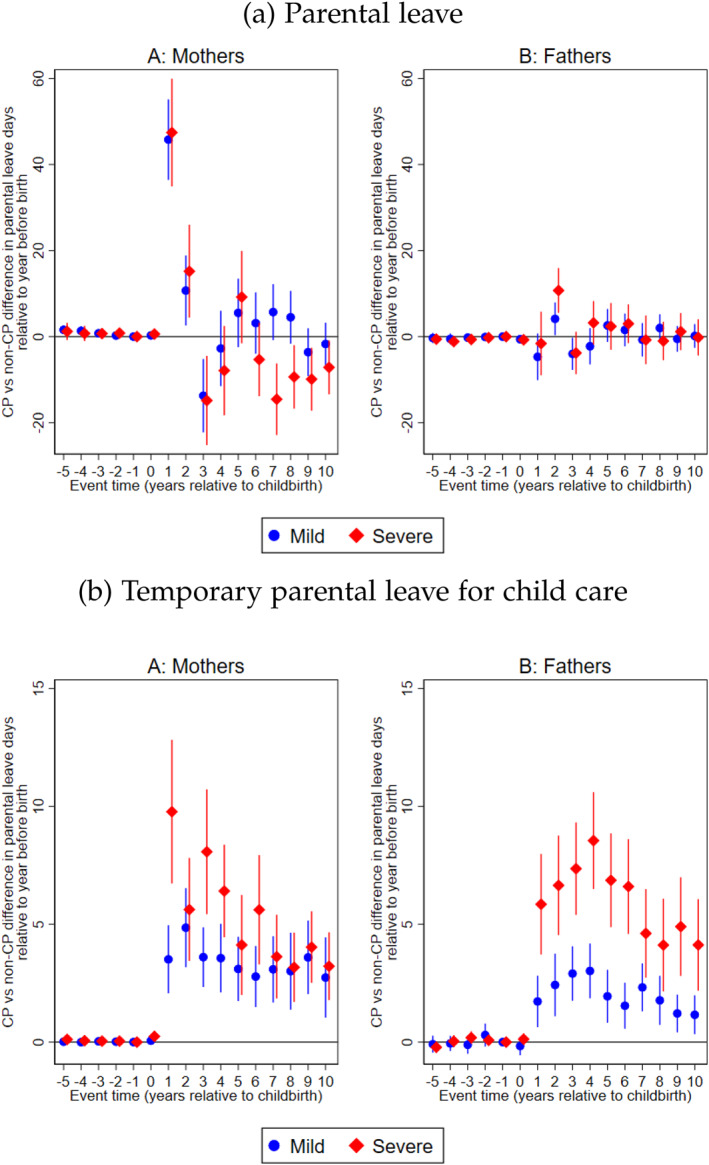
Days of parental leave in response to having a child with CP. The figure shows the trajectories of number of parental leave days for mothers and fathers, and 95% confidence intervals. Event time = 0 is the year of birth of the child.

We also examine parental outcomes such as disposable income (Figure A2), social insurance benefits such as income from parental leave and care allowance (Figure A3) and number of days of parental leave (Figure A4). For example, we find that care allowance to mothers of children with mild impairments increases from SEK 7200 (EUR 720) in the first year to SEK 30,000 (EUR 3000) by the 10th year. For mothers of children with severe impairments, care allowance increases from SEK 23,500 (EUR 2350) in the first year to SEK 51,000 (EUR 5100) in the third year and declines to SEK 27,000 (EUR 2700) by the 10th year.

#### 5.2.2 Parental Educational Attainment

In addition to severity of impairments, parental response to having a child with CP may vary across parental characteristics such as educational attainment. In addition, parents with higher socioeconomic status may have access to resources to cope with the care demands of having a child with CP. For example, parents with higher education may be employed in occupations with greater flexibility than parents with lower education. The application process for some disability‐related benefits such as personal assistants may be cumbersome and complex (Alriksson‐Schmidt et al. 2021), and higher educated parents may be more able to argue, articulate, and substantiate the needs of their child (Stormacq et al. 2019). In a recent study, Lichtenberg (2024) documents inequalities related to parental education in access to personal assistant services. To this end, we proceed to examine heterogeneity in parental labor‐market outcomes across parental educational attainment measured in the year prior to the birth of the child. We group parents into lower educated if their highest educational attainment is mandatory or secondary education and higher educated if they have completed higher education.

**FIGURE A5:**
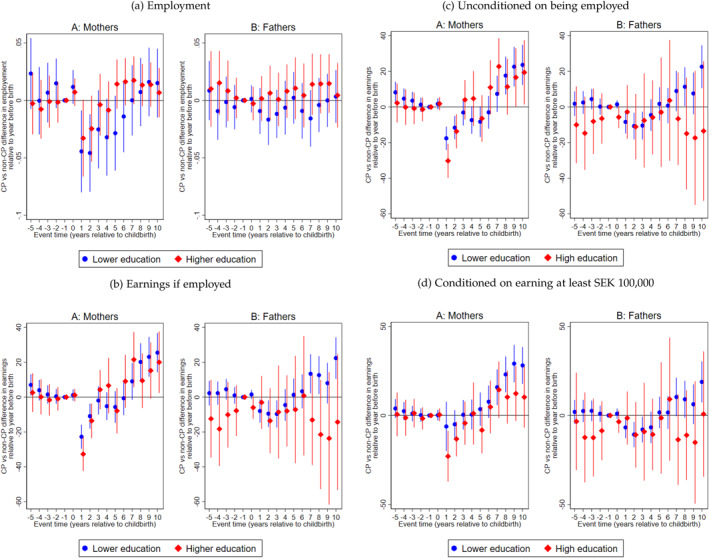
Parental labor‐market response to having a child with CP. The figure shows the trajectories of the employment and annual earnings conditioned on being employed during the year for mothers and fathers, and 95% confidence intervals. Event time = 0 is the year of birth of the child.

**FIGURE A6:**
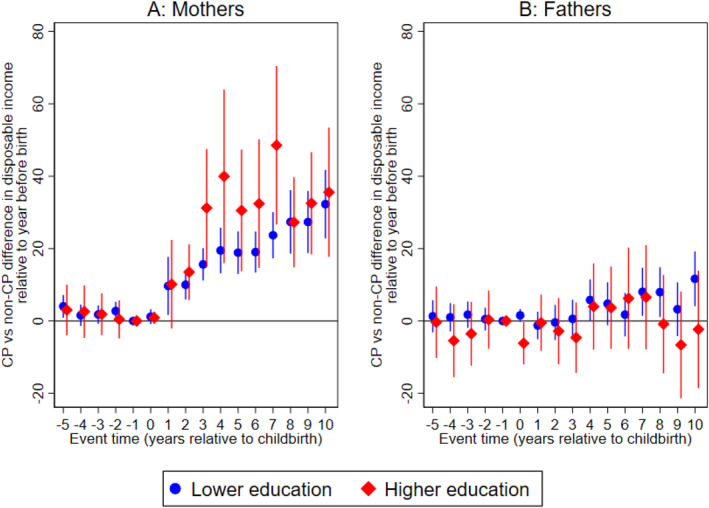
Parental disposable income response to having a child with CP. The figure shows the trajectories of disposable income mothers and fathers, and 95% confidence intervals. All monetary values are reported in 1000 SEK. Event time = 0 is the year of birth of the child.

**FIGURE A7:**
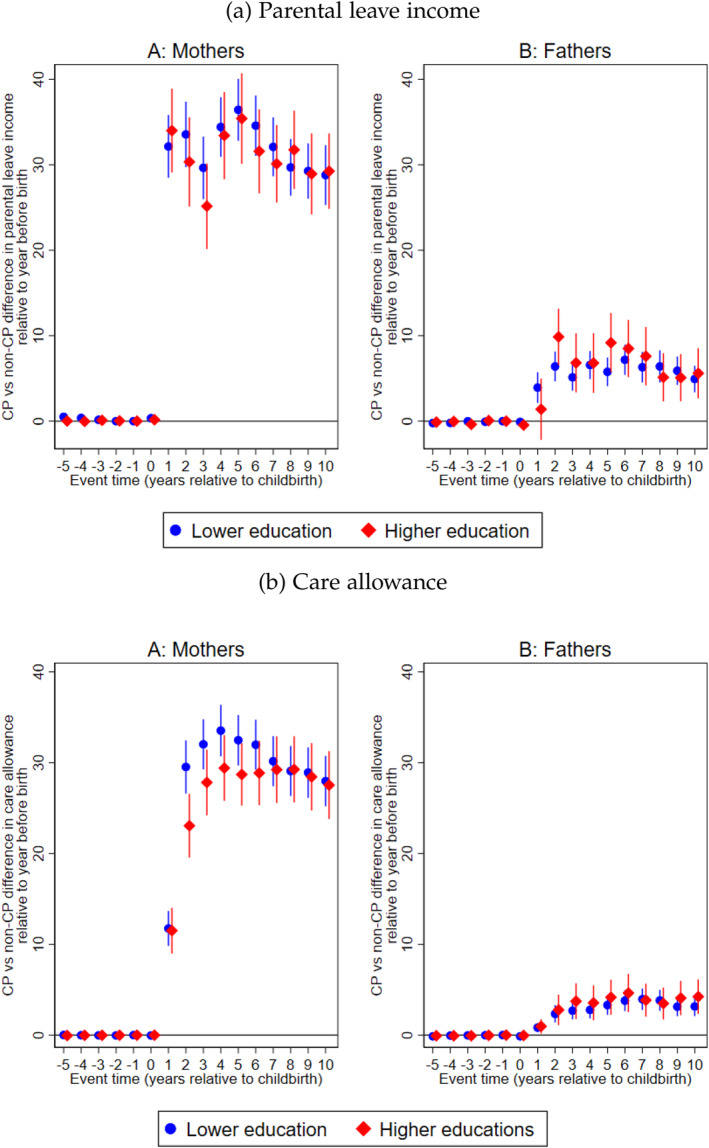
Benefits and transfers response to having a child with CP. The figure shows the trajectories of parental leave income and care allowance for mothers and fathers, and 95% confidence intervals. All monetary values are reported in 1000 SEK. Event time = 0 is the year of birth of the child.

**FIGURE A8:**
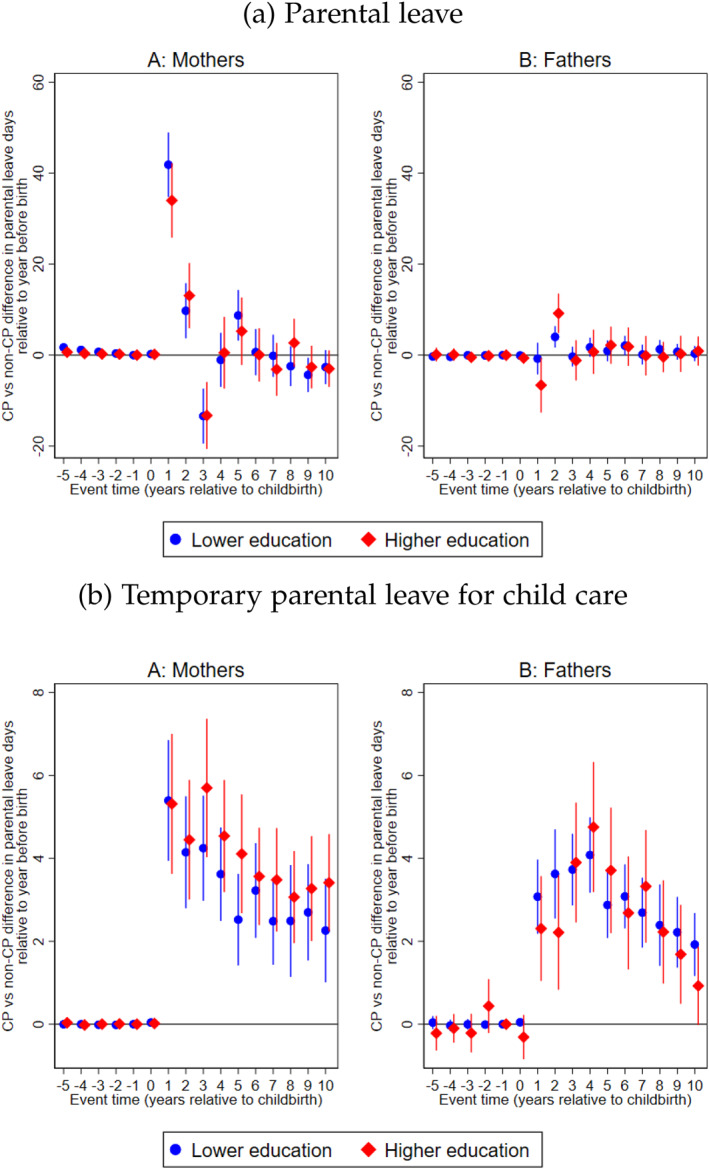
Days of parental leave in response to having a child with CP. The figure shows the trajectories of number of parental leave days for mothers and fathers, and 95% confidence intervals. Event time = 0 is the year of birth of the child.

We present the results in Appendix A as Figures A5, A6, A7, A8. Our results suggest that across most of the main labor‐market outcomes, maternal responses do not differ across educational attainment. For fathers, however, earnings shows a divergence starting from the seventh year after the child's birth. While the earnings of lower educated fathers show an upward trend, the earnings of higher educated fathers show a downward trend.

### 5.3 Potential Mechanisms

We explore potential mechanisms through which having a child with CP may affect parental labor‐market outcomes. To do this, we re‐estimate Equation (1) using outcomes variables that may mediate the impact of having a child with CP on parents' labor‐market outcomes, such as obtaining higher educational qualifications, job/occupational change, long‐term sickness leave, and subsequent childbirth.

**FIGURE A10:**
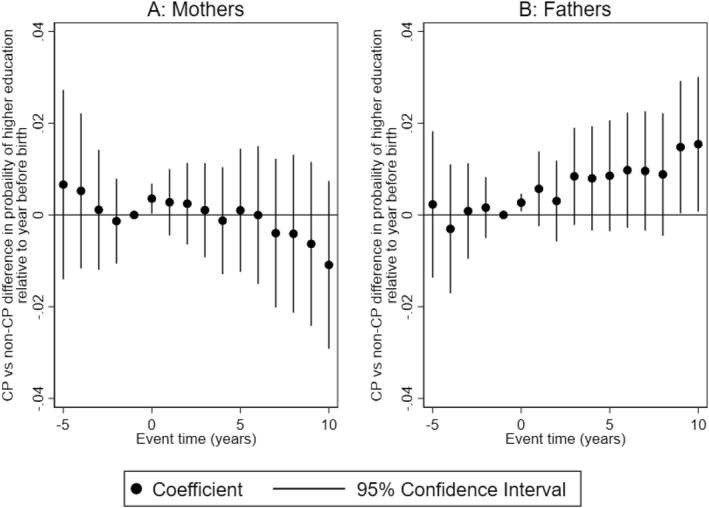
Parental probability of obtaining higher education in response to having a child with CP. The figure shows the estimates the probability of obtaining higher education for mothers and fathers, and 95% confidence intervals. Event time = 0 is the year of birth of the child.

One potential response for parents is to change their career trajectories by obtaining further education. Higher education improve earnings potential, as well as facilitates job changes and promotion into positions with family friendly working conditions. In Figure A10, we observe that the likelihood of obtaining higher education differs between mothers and fathers in the treated group, thought not statistically significant. While the likelihood of treated mothers to obtain higher education decreases, the likelihood increases for treated father.

**FIGURE A11:**
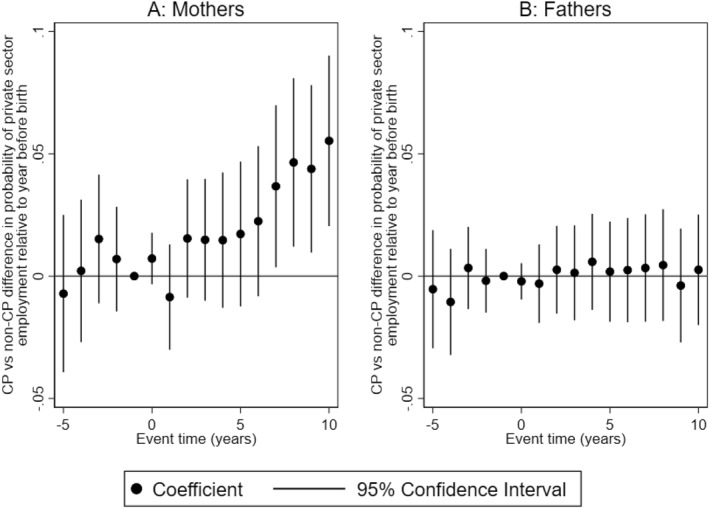
Parental probability of working private sector if employed in response to having a child with CP. The figure shows the estimates the probability of working in at private sector if employed with a strong attachment to the labor market for mothers and fathers, and 95% confidence intervals. Event time = 0 is the year of birth of the child.

Parents may choose jobs that offer greater work flexibility in response to having a child with a disability (Eriksen et al. 2021). The public sector is particularly well known for such work flexibility (Kleven et al. 2019), but also pay less compared to the private sector. However, parents may also choose to go into self‐employment or take up jobs in the private sector as the personal assistant to the child with CP. We find that treated mothers are 4%–5% points more likely to work in the private sector conditioned on being employed with a strong attachment to the labor market in the long‐run, compared to mothers in the comparison group (see Figure A11). We find no effect on the sector of employment of fathers. This finding may suggest that mothers of children with CP take up jobs as personal assistants to their child in the private sector or engage in self‐employed activities for the flexibility to combine labor‐market engagement with caregiving responsibilities.

**FIGURE A12:**
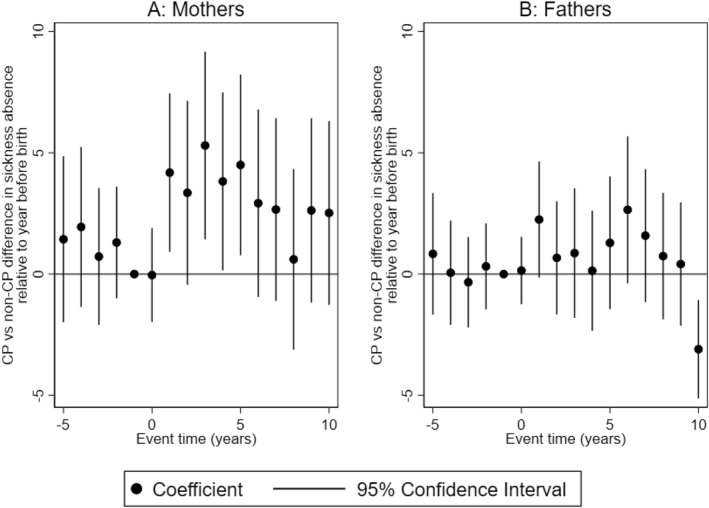
Parental number of days of sickness leave in response to having a child with CP. The figure shows the estimates the number of days of sickness leave for mothers and fathers, and 95% confidence intervals. Event time = 0 is the year of birth of the child.

The demands of caring for a child with CP may lead to fatigue, stress and pain that may increase work absenteeism. In Sweden, employers pay for the first 14 days of a sick‐leave spell, after which additional days are covered by the social insurance system. Our data captures long‐term sickness absence, defined as work absence exceeding the first 14 days. Treated mothers take more days on long‐term sickness leave than mothers in the comparison group, though the difference is statistically significant only in the third and fifth years with an increase of 4 days (see Figure A12).

**FIGURE A13:**
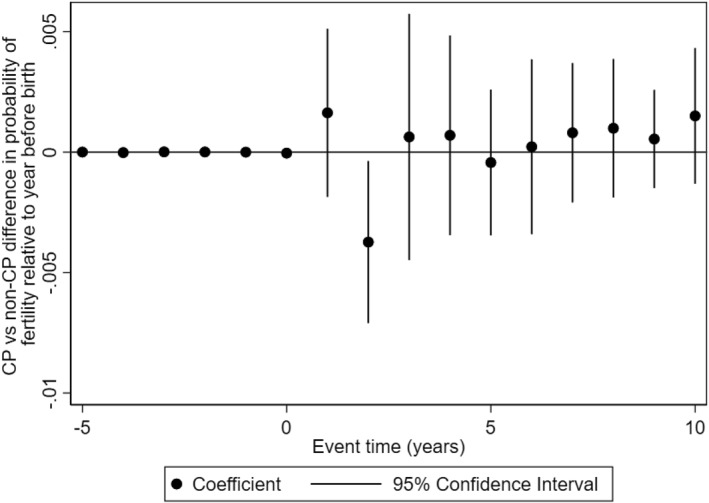
Maternal probability of subsequent childbirth in response to having a child with CP. The figure shows the estimates the probability of subsequent childbirth for mothers and 95% confidence intervals. Event time = 0 is the year of birth of the child.

Previous research (Müller et al. 2022) finds that parents of children with CP are less likely to have additional children. For mothers, we investigate whether subsequent childbirth is a potential mediator between having a child with CP and labor‐market outcomes. Using the Medical Birth Register, we are able to identify mothers who gave birth within the Swedish healthcare system. We find that the probability of having additional children is reduced for treated mothers in the second year after the birth of the child (see Figure A13).

**FIGURE A14:**
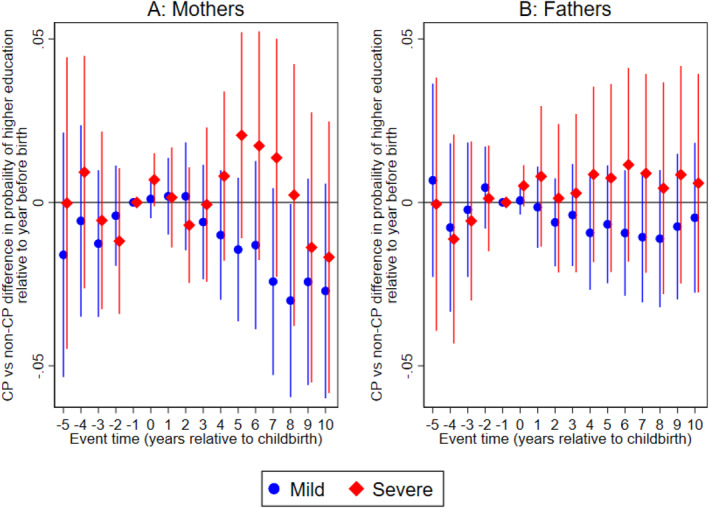
Parental probability of obtaining higher education in response to having a child with CP. The figure shows the estimates the probability of obtaining higher education for mothers and fathers, and 95% confidence intervals. Event time = 0 is the year of birth of the child.

**FIGURE A15:**
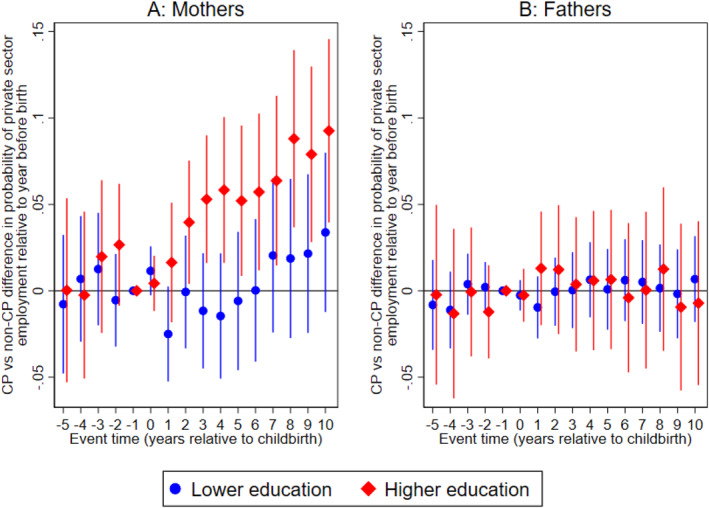
Parental probability of working private sector if employed in response to having a child with CP.The figure shows the estimates the probability of working in at private sector if employed for mothers and fathers, and 95% confidence intervals. Event time = 0 is the year of birth of the child.

**FIGURE A16:**
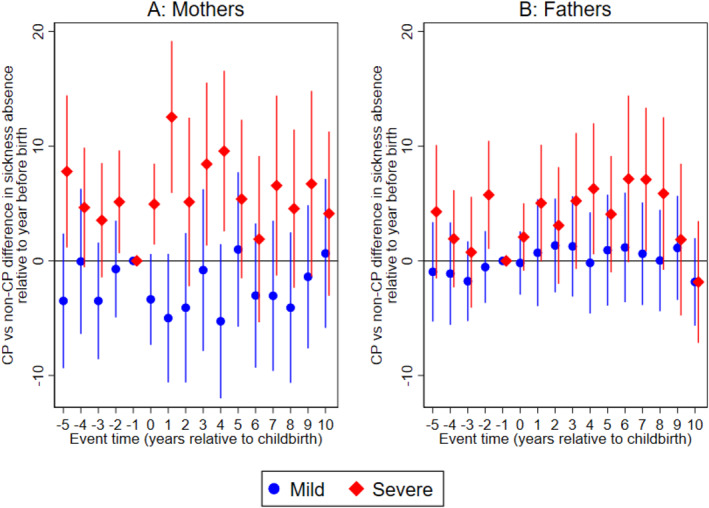
Parental number of days of sickness leave in response to having a child with CP. The figure shows the estimates the number of days of sickness leave if employed for mothers and fathers, and 95% confidence intervals. Event time = 0 is the year of birth of the child.

**FIGURE A17:**
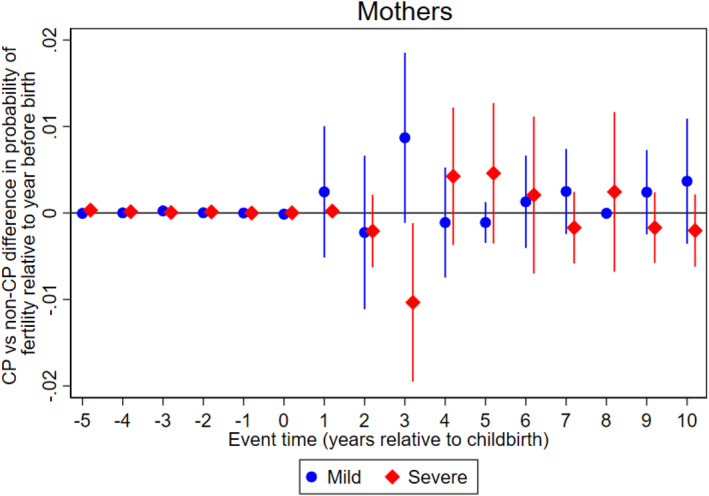
Maternal probability of subsequent childbirth in response to having a child with CP. The figure shows the estimates the probability of subsequent childbirth for mothers and 95% confidence intervals. Event time = 0 is the year of birth of the child.

**FIGURE A18:**
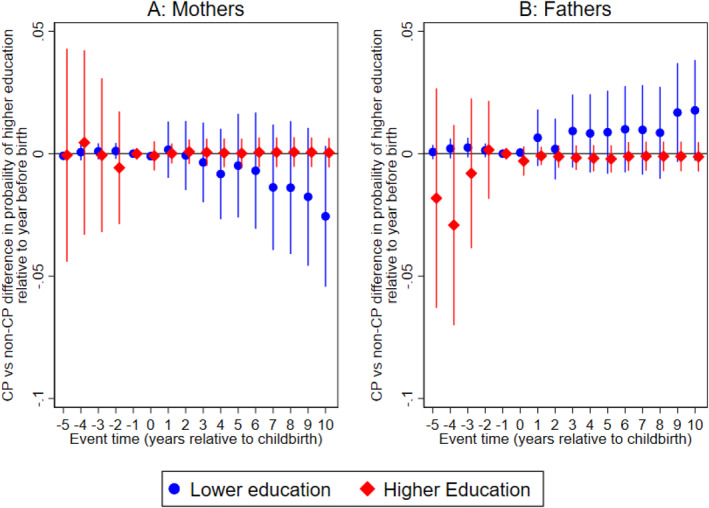
Parental probability of obtaining higher education in response to having a child with CP. The figure shows the estimates the probability of obtaining higher education for mothers and fathers, and 95% confidence intervals. Event time = 0 is the year of birth of the child.

**FIGURE A19:**
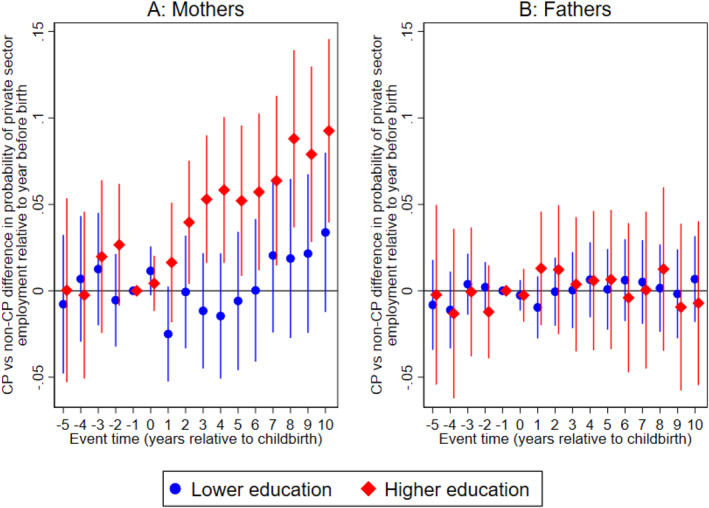
Parental probability of working private sector if employed in response to having a child with CP. The figure shows the estimates the probability of working in at private sector if employed for mothers and fathers, and 95% confidence intervals. Event time = 0 is the year of birth of the child.

**FIGURE A20:**
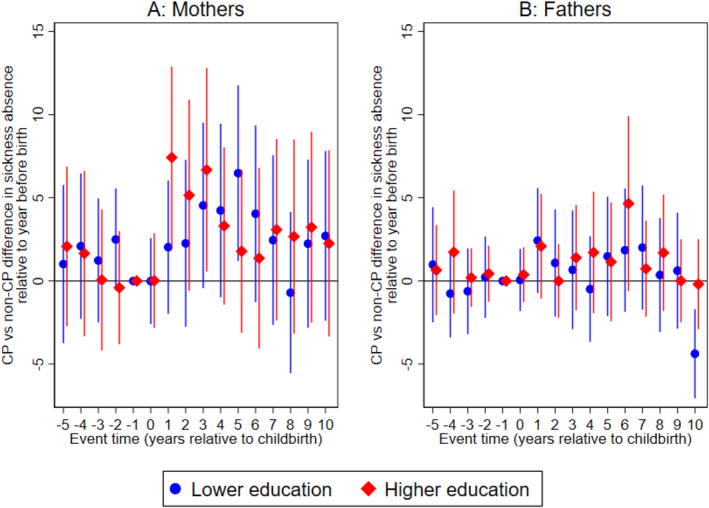
Parental number of days of sickness leave in response to having a child with CP. The figure shows the estimates the number of days of sickness leave if employed for mothers and fathers, and 95% confidence intervals. Event time = 0 is the year of birth of the child.

**FIGURE A21:**
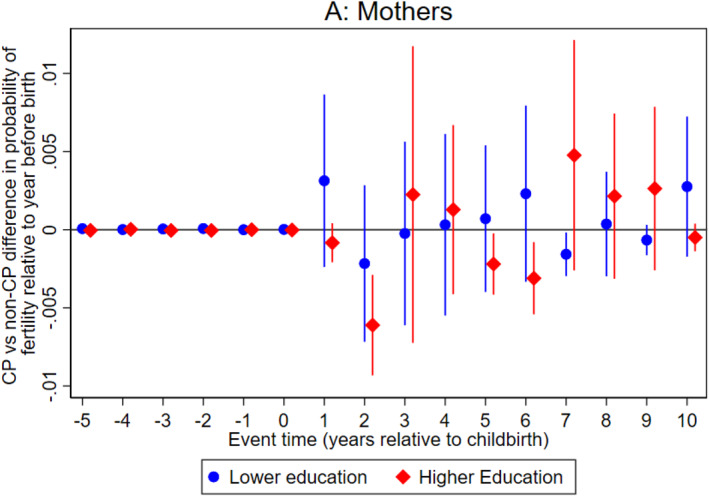
Maternal probability of subsequent childbirth in response to having a child with CP. The figure shows the estimates the probability of subsequent childbirth for mothers and 95% confidence intervals. Event time = 0 is the year of birth of the child.

We also examine whether the mechanisms underlying the impact of child disability on parental labor‐market outcomes differ by severity of impairment and parental educational attainment. Detailed results are available in the Appendix as Figures A14, A15, A16, A17, A18, A19, A20, A21. Our results show variations across both severity of impairments and parental educational attainment. Mothers of children with severe impairments, and higher education are more likely to work in the private sector following the birth of a child with CP. Parents of children with severe impairments take more days of sickness leave compared to parents of children with mild impairments. Finally, the likelihood of having additional children differ by severity of impairment. Mothers of children with severe impairment are less likely to have additional children by the third year following the birth of the first child, compared to mother of children with mild impairment.

## 6 Discussion

In this paper, we estimate the dynamic impact of having a child with an early‐onset disability on parental labor‐market outcomes. While most existing studies treat childhood disabilities as a broad and heterogeneous group, specific disabilities manifest in different ways, necessitating a more focused approach to improve policy responses. We therefore focused on Cerebral Palsy (CP) and contribute to the growing literature on the impact of specific early‐onset disabilities on parental labor market trajectories.

We leverage rich Swedish administrative data and several features of CP onset, employing an empirical strategy that allows for causal interpretation. Our analysis compares parents of first‐born children with CP to parents of children without CP from a matched control group. Although, the exact causes of CP remain uncertain, we show that having a child with CP is uncorrelated with parental characteristics in the year preceding the child's birth. Further, the wide variation in functioning and severity among children with CP enables us to assess the impact across CP severity levels and examine parents' use of the social insurance system in Sweden.

We find that the impact of having a child with CP differ between fathers and mothers. In the short‐run, employment and earnings of mothers are negatively impacted by the birth of a child with CP, whereas the impact on fathers is small. These short‐run effects are similar to other studies in the field, such as Adhvaryu et al. (2023) and Breivik and Costa‐Ramón (2024). In the long‐run however, the employment of treated mothers return to their pre‐birth levels whilst earnings increase compared to mothers in the comparison group. These long‐run effects are contrary to Gunnsteinsson and Steingrimsdottir (2019) and Adhvaryu et al. (2023) who report negative long‐run consequences in Denmark of having a child with CP and cancer respectively. On the other hand, the results are similar to Öhman et al. (2021) who find a positive trajectory for maternal earnings following the child being diagnosed with cancer in Sweden.

Further, we find that treated mothers are more likely to be employed in the private sector, which is contrary to Eriksen et al. (2021) who report that mothers of children with early‐onset type‐1 diabetes are more likely to be employed in the public sector in Denmark. The increase in the likelihood to be employed in the private sector is driven by mothers of children with severe impairments. This could be due to mothers shifting from public sector jobs to self‐employment or taking up positions as personal assistants to their children with private sector agencies. Self‐employment provides caregivers the flexibility to combine childcare responsibilities with active labor market participation. The absence of an impact on fathers indicate that parents choose to leave fathers' employment unaffected, potentially for economic (men tend to earn more than women (Swedish National Mediation Office 2023)) or cultural reasons. However, it should be noted that the differences between mothers and fathers could be driven by the fact that while we focus on first births for mothers, we are unable to identify the birth order for fathers.

We also find that the Swedish social welfare system compensates parents through transfers such as parental leave income and care allowance. However, as we do not have data on the specific expenditures associated with the disability, we cannot estimate the extent of compensation. The difference in disposable income prompted by parental benefits and care allowance between mothers and fathers of children with CP may reflect the gendered division of labor where women take a larger responsibility in childcare. During the period of this study, care allowance was paid to the parent who was the primary caregiver of the child within the household.

Parents may increase their labor supply in response to the financial burdens that having a child with a disability may impose (Gould 2004). However, our results must be understood within the context of the Swedish welfare state. In Sweden, healthcare is tax funded with universal access to services, treatments, medications and assistive devices. As such, the long‐run increase in maternal employment cannot be driven by health insurance motives, which may be the case in countries where the cost of healthcare are borne by parents or where health insurance is tied to parental employment status (Wasi et al. 2012; Eriksen et al. 2021).

The increases in maternal employment and earnings are primarily driven by mothers of children with severe impairments. This contrast with to Wasi et al. (2012) who find that severe childhood disabilities lead to large reductions in maternal labor supply. The difference between our findings and those of Wasi et al. (2012) likely reflects differences in the social settings of the studies. Whilst Wasi et al. (2012) are situated in the US where the social support system for parents of children with disabilities may not be extensive, parents of children with disabilities in Sweden are entitled to several benefits from the social insurance system that are expected to affect their labor‐market participation and outcomes, such as personal assistance for their children with disabilities.

Our empirical approach rests on the assumption that parental characteristics of children with and without CP was similar before the birth of the child. Although we argue that this assumption is plausible in the Swedish context based on previous studies, this might not hold for age, employment (mothers), and education (fathers), indicating a potential socioeconomic gradient. Most likely, this would exaggerate the impact of having a child with CP on parental outcomes. However, the relatively small differences and the results of the multiple hypothesis testing indicating the absence of significant differences, do suggest that differences in parental characteristics before birth of the child are random. In addition, there is little evidence on if parental characteristics predict level of severity of CP (instead of onset), although it is presumably related to severity of secondary conditions. As we define severity based on GMFCS level and CP‐subtype, we consider it unlikely that any significant selection exists, although the possibility cannot be rejected. It should also be noted that our control group is based on the general population and may therefore include parents of children with other forms of disabilities. However, this should not bias our estimates. Persons with CP may have other disabilities, including intellectual (Reid et al. 2018) and neuro‐psychiatric ((Påhlman et al. 2019)) disorders that may not be the result of the initial brain damage. As such, comparing parents of children with CP to the general population enables us to understand the labor‐market consequences of having a child with CP compared to the average population.

The difference in our findings to other studies such as those by Eriksen et al. (2021), Gunnsteinsson and Steingrimsdottir (2019) and Adhvaryu et al. (2023) that use data from Danish administrative registers may in part be explained by the differences in social insurance systems between Sweden and Denmark. While both countries have extensive social protection initiatives for children with disabilities and their families, there exist substantial differences, particularly in the provision of personal assistance for children with disabilities. Unlike Sweden, the Danish social insurance system does not provide paid personal assistants to persons with disabilities who are less than 18 years old. However, parents in Denmark may be compensated for lost earnings, to a capped amount, if the parent must reduce their labor‐market activities due to the disability of the child.

Access to personal assistance can affect parental labor‐market outcomes through two possible channels. Personal assistants free up parental time and thereby increase opportunities for labor market activities. Parents also have the option to take up a position as a paid personal assistant to their child (Eriksen et al. 2021), or work extra hours in addition to their normal employment. This channel is expected to have been reinforced by a shortage of suitable personal assistants (Ahlström and Wadensten 2011). Further, persons with severe disabilities are more likely to be granted personal assistants von Granitz et al. (2017), which suggests that the difference in labor‐market outcomes between parents of children with mild and severe impairments may be driven by the differences in access to personal assistance. The salary for personal assistants ranges between SEK 27,000‐30,000. Thus, the long‐term earnings increase among mothers corresponds to less than 1 month full time equivalent as a personal assistant. Unfortunately, our data do not allow us to identify whether a parent of the child is employed as the personal assistant, the number of hours worked, or any potential reduction in work hours in the ”main” employment. It is therefore not possible to determine the precise mechanism of the earnings effect in the current study.

While we observe long‐run increases in maternal labor‐market outcomes, these do not necessarily translate to better career trajectories. In fact, it may be the opposite. Caring for a child with CP often involves strenuous activities such as feeding the child through a tube or lifting the child periodically to avoid pressure injuries. If parents of children with CP combine regular employment with responsibilities of care‐giving, they are likely to experience stress and physical and mental fatigue and, in extension, poor mental health and low quality of life (Aktan et al. 2020; Farajzadeh et al. 2020; Fritz and Sewell‐Roberts 2020; Asuman et al. 2025). Moreover, the role of that of a parent is different from that of a personal assistant, and at least anecdotally, some parents express a wish to be “just a parent”. Also, working as a personal assistant and also being the parent of a child with severe disability might result in social isolation, as it might hinder the interaction with other adults. Thus, the long‐run gains in labor‐market outcomes must be understood alongside possible negative consequences on family‐work balance, life satisfaction, and mental and physical health (Skoy 2024), which warrants future research.

Our results show that the effects of having a child with CP is strongest for women which is consistent with the findings of Gunnsteinsson and Steingrimsdottir (2019), Adhvaryu et al. (2023) and Eriksen et al. (2021), and that highlights the persistent gendered division of caregiving responsibilities. Despite Sweden's egalitarian family model, significant gender gaps in parental and household duties remain (Moberg and van der Vleuten 2022). These findings emphasize the need for further studies on the role of social insurance system, particularly access to personal assistants, in shaping parental labor‐market outcomes in Sweden.

## Conflicts of Interest

The authors declare no conflicts of interest.

## Figures and Tables

**FIGURE A9:**
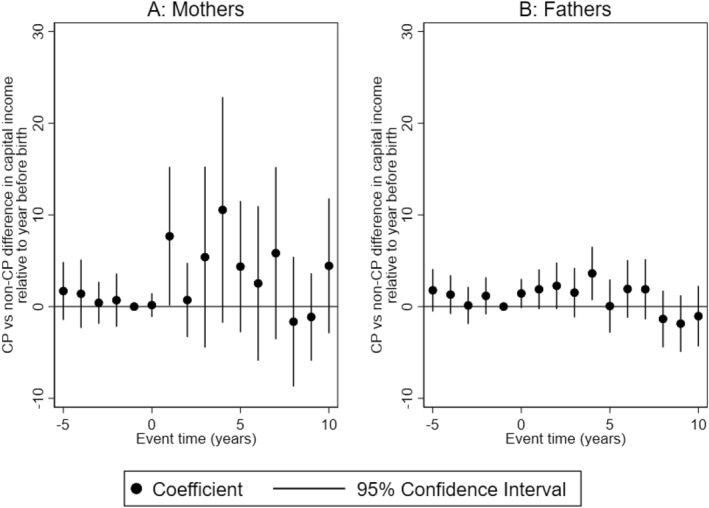
Parental capital income response to having a child with CP. The figure shows the trajectories of capital income for mothers and fathers, and 95% confidence intervals. All monetary values are reported in 1000 SEK. Event time = 0 is the year of birth of the child.

## Data Availability

Deidentified individual participant data will not be made available by the authors but are available from the register holders after typical application procedures.

## Endnotes

^1^

https://www.who.int/health‐topics/congenital‐anomalies#tab=tab_1.

^2^
ICD10: G00, S061, S062, S063, S067, S068, S069.

^3^
ICD10: G60, G61, G62, G71, G72, G834, G95, E71, E72, E74, E75, E76, E830, G114, G12, G31, G37, Q06, Q743.

^4^
SEK 10 ≈ EUR 1 in 2015.

^5^
Average earnings conditioned on being employment for mothers in the control was SEK 199,920 (EUR 19,992) in the reference year.

^6^
Average earnings conditioned on being employment for fathers in the control was SEK 261,460 (EUR 26,146) in the reference year.

^7^
Average earnings unconditioned on being employment for mothers in the control was SEK 189,310 (EUR 18,931) in the reference year.

^8^
Average earnings unconditioned on being employment for fathers in the control was SEK 246,240 (EUR 24,624) in the reference year.

^9^
Average earnings conditioned on on earning at least SEK 100,000 for mothers in the control was SEK 236,170 (EUR 23,617) in the reference year.

^10^
Average earnings conditioned on on earning at least SEK 100,000 for fathers in the control was SEK 298,740 (EUR 29,874) in the reference year.

^11^
We also examine the impact of having a child with CP on parental capital income but find no significant effects (Figure A9).
